# Rosiglitazone suppresses RANKL‐induced NFATc1 autoamplification by disrupting the physical interaction between NFATc1 and PPARγ

**DOI:** 10.1002/2211-5463.12513

**Published:** 2018-09-10

**Authors:** Kyeong‐Lok Park, Da‐Gyo Oh, Young‐Ok Kim, Kyeong‐Seob Song, Do‐Whan Ahn

**Affiliations:** ^1^ Department of Dentistry Kosin University Gospel Hospital Seo‐gu Korea; ^2^ Department of Physiology Kosin University College of Medicine Seo‐gu Korea; ^3^ Department of Pathology Kosin University College of Medicine Seo‐gu Korea

**Keywords:** NFATc1, osteoclastogenesis, physical interaction, PPARγ, rosiglitazone

## Abstract

Receptor activator of nuclear factor‐κB ligand (RANKL) is required for initiation of osteoclastogenesis, with the signaling pathway including the NF‐kB, c‐Fos, and nuclear factor of activated T cells, cytoplasmic 1 (NFATc1) transcription factors. Because NFATc1 expression is autoamplified, we investigated the molecular mechanism by which peroxisome proliferator‐activated receptor gamma (PPARγ) activation by the thiazolidinedione drug rosiglitazone decreases NFATc1 expression during RANKL stimulation. Western blotting demonstrated that rosiglitazone attenuated the increase in NFATc1 protein level induced by RANKL without affecting that of PPARγ. Immunofluorescence data indicated that rosiglitazone tended to suppress RANKL‐induced NFATc1 nuclear translocation, partly by reducing calcineurin activity, as reflected by the observed decrease in nuclear NFATc1 abundance. On coimmunoprecipitation, the intensity of the physical interaction between NFATc1 and PPARγ was unexpectedly higher in the RANKL‐stimulated group than in the control, but rosiglitazone reduced this to basal levels. Furthermore, RANKL failed to elevate mRNA expression of *NFATc1* after PPARγ knockdown. ChIP assay indicated that rosiglitazone significantly reduced the binding of NFATc1 to its own promoter despite RANKL stimulation. These findings suggest that PPARγ activation by rosiglitazone blocks NFATc1 from binding to its own promoter, thereby reducing RANKL‐induced NFATc1 autoamplification.

**Abbreviations**
AP‐1: activator protein‐1
ChIP: chromatin immunoprecipitation
FBS: fetal bovine serum
MAPK: mitogen‐activated protein kinase
NFATc1: nuclear factor of activated T cells, cytoplasmic 1
PPARγ: peroxisome proliferator‐activated receptor gamma
RANKL: nuclear factor‐κB ligand
RANK: receptor activator of NF‐κB
TRAP: tartrate‐resistant acid phosphatase

The formation and differentiation of osteoclasts occur through tightly regulated processes. In general, initiation of osteoclastogenesis requires receptor activator of nuclear factor‐κB ligand (RANKL). RANKL signaling recruits multiple intracellular signaling molecules, of which the NF‐kB, c‐Fos, and nuclear factor of activated T cells, cytoplasmic 1 (NFATc1) transcription factors are important for osteoclastogenesis both *in vivo* and *in vitro* 1, 2. Previous studies have clearly shown that NFATc1‐deficient stem cells or preosteoclasts fail to differentiate into osteoclasts 3, 4, 5, and conditional or rescued NFATc1‐deleted mice develop severe osteopetrotic phenotypes 5, 6. Furthermore, NFATc1 directly regulates osteoclast‐specific genes such as tartrate‐resistant acid phosphatase (*TRAP*), calcitonin receptor, cathepsin K, and β3‐integrin 7, 8, 9, thereby underscoring that NFATc1 is a master regulator of osteoclastogenesis. Because NFATc1 expression undergoes positive autoregulatory feedback 4, 10, 11, 12, NFATc1 nuclear translocation is necessary to initiate this step. Within the nucleus, NFATc1 binds to several sets of NFAT‐binding motifs in its own promoter. When it binds to the promoter, NFATc1 forms heterodimers with other transcription factors, including peroxisome proliferator‐activated receptor gamma (PPARγ), activator protein‐1 (AP‐1), and NF‐κB 13. Accordingly, these transcription factors can play specific roles in modulating the transcriptional activity of NFATc1.

Peroxisome proliferator‐activated receptor gamma is a nuclear receptor transcription factor critical for adipocyte differentiation and is also involved in bone homeostasis and remodeling. Activation of PPARγ by thiazolidinedione drugs such as rosiglitazone and pioglitazone increases bone loss in normal mice and fracture incidence in diabetic humans 14, 15, implying that PPARγ may regulate the function or formation of both osteoblasts and osteoclasts. Although several reports suggested that PPARγ activation by thiazolidinedione activates RANKL‐induced osteoclastogenesis 16, 17, many *in vitro* studies have demonstrated that PPARγ activation inhibits it 18, 19, 20, 21, 22.

The molecular mechanisms by which PPARγ activation inhibits osteoclastogenesis vary from reduction in NF‐κB expression, suppression of NF‐κB nuclear translocation and its DNA binding, and downregulated expression of c‐Fos, to inhibition of mitogen‐activated protein kinase activity 19, 21, 23. Although PPARγ activation also reduces NFATc1 expression at the transcriptional and protein levels during RANKL‐induced osteoclastogenesis, the mechanism by which PPARγ activation decreases NFATc1 expression has not been investigated. We explored the molecular mechanisms by which PPARγ activation by rosiglitazone suppresses NFATc1 expression during the early period of RANKL‐induced osteoclastogenesis using RAW264.7 monocytes.

## Materials and methods

### Cell culture and drug treatment

RAW264.7 cells were routinely cultured in α‐MEM (Gibco, Waltham, MA, USA) supplemented with 10% FBS (Gibco) and 1% penicillin–streptomycin at 37 °C in a 5% CO_2_ condition. Rosiglitazone (Cayman Chemical, Ann Arbor, MI, USA) was added to the medium 30 min before RANKL (R&D Systems, Minneapolis, MN, USA) administration, and cells were maintained for an additional 24 h. For siRNA experiments, complete DMEM/F12 (Gibco) without antibiotics was used as the culture medium.

### Osteoclastogenesis and cell viability

To observe osteoclast differentiation, RAW264.7 cells were cultured in 96‐well plates in medium containing various concentrations of rosiglitazone in the presence 50 μg·mL^−1^ of RANKL. After 5 days, cells were stained for TRAP with a commercial kit (TRACP & ALP double‐stain kit, Takara, Shiga, Japan). Cytotoxicity of rosiglitazone was assayed using an MTT reagent (Amresco, Inc., Solon, OH, USA). In this case, cells were treated for 1 or 5 days without RANKL addition.

### Conventional RT‐PCR

Total RNA was extracted using TRIzol reagent (Invitrogen, Carlsbad, CA, USA), and 1 μg of RNA was used as the template to synthesize cDNA using AccuPower^®^ CycleScript RT PreMix kit (Bioneer, Daejeon, Korea). The end‐point RT‐PCRs were carried out using AccuPower^®^ HotStart PCR PreMix kit (Bioneer). Primer sequences were as follows: NFATc1 (300 bp): forward, TGC TCC TCC TCC TGC TGC TC, reverse, CGT CTT CCA CCT CCA CGT CG; TRAP (275 bp): forward, GAT GAC TTT GCC AGT CAG CA, reverse, ACA TAG CCC ACA CCG TTC TC; receptor activator of NF‐κB (RANK; 255 bp): forward, CTG CCT GAA ATG TG ACC AT, reverse, TGG CTG ACA TAC ACC ACG AT; β‐actin (216 bp): forward, GAC GGC CAG GTC ATC ACT AT, reverse, CTT CTG CAT CCT GTC AGC AA.

### Western blotting

Whole cell lysates or immunoprecipitated complexes were prepared in RIPA buffer (Thermo Fisher Scientific, Inc., Rockford, IL, USA) with 1x protease inhibitor cocktail (Sigma‐Aldrich, St. Louis, MO, USA). Nuclear and cytoplasmic extracts were prepared using a commercial kit (G‐Bioscience, St. Louis, MO, USA). Immunoblots were performed by routine procedures as described previously 24. Blots were probed with mouse monoclonal anti‐NFATc1(1 : 1000, Santa Cruz, Dallas, TX, USA) and rabbit polyclonal anti‐NFATc1 (1 : 1000, Santa Cruz), mouse monoclonal anti‐PPARγ (1 : 1000, Santa Cruz) and rabbit polyclonal anti‐PPARγ (1 : 1000, Santa Cruz), rabbit polyclonal anti‐calcineurin Aα (catalytic subunit A isoform alpha; 1 : 3000, Santa Cruz) and anti‐calcineurin B2 (regulatory subunit B type 2; 1 : 3000, Flarebio, College Park, MD, USA), mouse monoclonal anti‐lamin A/C (1 : 3000, Cell Signaling, Danvers, MA, USA), or rabbit polyclonal anti‐β‐actin (1 : 5000, Cell Signaling).

### Calcineurin activity

Cellular calcineurin (PP2B) activity was measured using a kit according to the manufacturer's instructions (Enzo Life Sciences, Farmingdale, NY, USA). Cells incubated in six‐well plates were lysed in lysis buffer. Cell lysate (20 μg protein) from each group or a positive calcineurin (40 U) control was added to the tube with or without EGTA buffer, and the reaction with phosphopeptide substrate was conducted at 30 °C for 30 min and terminated by adding stop buffer. The phosphate standard curve was prepared by serial dilution of phosphate solution. Because calcineurin is a Ca^++^/calmodulin‐dependent phosphatase, its activity was determined by subtracting Ca^++^‐independent activity in the presence of EGTA from total activity in the absence of EGTA. Final calcineurin activity was presented as the amount of inorganic phosphate released per μg of protein. In our experiment, the presence of EGTA quenched total calcineurin activity by approximately 60–70%.

### Immunofluorescence

Immunofluorescent detection of NFATc1 was performed according to the method described by Hasega *et al*. 25 with minor modifications. Briefly, cells were plated at less than 50% confluence (a density of 5 × 10^4^ cells/chamber) in a four‐chamber slide (Nunc, Rochester, NY, USA), grown overnight, and treated with vehicle or drugs for 24 h in complete α‐MEM. After fixation with 4% paraformaldehyde for 10 min at room temperature, cells were permeabilized with 0.2% Triton X‐100 for 15 min and then blocked with 5% bovine serum albumin at room temperature for 1 h. After washing and blocking, anti‐NFATc1 antibody was added at a dilution of 1 : 100 and the fixed cells were incubated at 4 °C overnight. NFATc1 signals were detected with Alexa Fluor 488‐conjugated secondary antibody (Invitrogen) at a dilution of 1 : 500 for 1 h, after which the fixed cells were mounted in DAPI‐containing medium (Sigma).

### Coimmunoprecipitation

Peroxisome proliferator‐activated receptor gamma or NFATc1 was immunoprecipitated by incubating whole cell lysates (200–300 μg of protein) with anti‐PPARγ or anti‐NFATc1 antibody overnight at 4 °C. NFATc1 immunocomplexes were captured with protein G beads (Invitrogen) for 1 h at 4 °C. PPARγ immunocomplexes were captured using the ImmunoCruz Optima C System (Santa Cruz) to avoid cross‐reaction with immunoglobulin heavy chain, which has a molecular weight similar to that of PPARγ. The resulting immunocomplexes were collected by centrifugation. After washing, the pellets were eluted and immunoblotted as described above.

### siRNA

Cells were seeded at less than 80% confluence in six‐well plates and cultured in antibiotic‐free DMEM/F12 with 10% FBS overnight. Transfection was carried out according to the manufacturer's protocol. SmartPool PPARγ siRNA or control siRNA (Dharmacon, Lafayette, CO, USA) was used at a final concentration of 100 nm using DharmaFECT 4 transfection reagent (Dharmacon). At 24 h after transfection, cells were treated with relevant drugs and cultured for an additional 24 h in DMEM/F12.

### ChIP assay

Cells were cultured in 100‐mm dishes and transfected with control siRNA or PPARγ siRNA. ChIP assays were performed with monoclonal anti‐NFATc1 or mouse IgG. ChIP procedures were performed according to the protocol provided by the supplier (Upstate Biotechnology, Lake Placid, NY, USA). Primer sequences for amplifying the region including the NFAT‐binding sites of the mouse *NFATc1* P1 promoter were published previously 11. The precipitated DNA was evaluated by quantitative PCR using Universal SYBR Green Master Mix (Takara, Shiga, Japan). *C*_t_ values were normalized to input using the comparative delta–delta method 26.

### Statistical analysis

Data are expressed as mean ± SD and were analyzed by either one‐way or two‐way ANOVA with the LSD test using IBM spss 23 (Armonk, NY, USA). *P*‐values < 0.05 were considered statistically significant.

## Results

### Effect of rosiglitazone on osteoclastogenesis and NFATc1 expression

**Figure 1:**
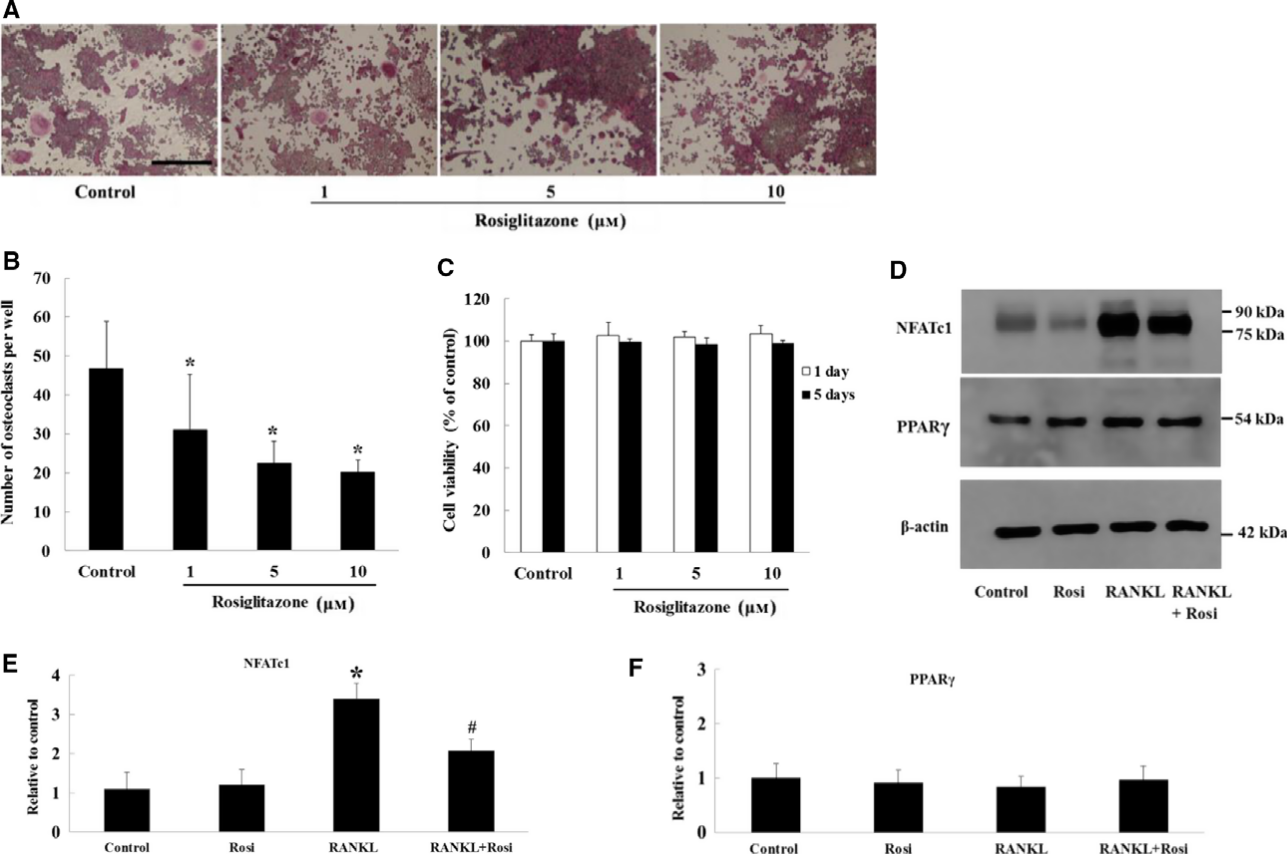
Rosiglitazone decreases RANKL‐induced osteoclastogenesis and NFATc1 expression. Cells were cultured in 96‐well plates and treated with vehicle or different doses of rosiglitazone for 5 days in the presence of RANKL (50 μg·mL^−1^). (A) A representative image of TRAP‐stained osteoclast differentiation is shown at × 100 magnification. Scale bar, 100 μm. (B) The number of TRAP‐positive cells with more than three nuclei was counted. Data were drawn from three wells of a single experiment. *P <* *0.05 vs. control. Rosi, rosiglitazone. (C) Cytotoxicity of rosiglitazone was determined using MTT assay. In this experiment, cells were treated with different doses of rosiglitazone for 24 h and 5 days in the absence of RANKL. (D) Cells were treated with vehicle or 5 μm of rosiglitazone for 24 h in the presence or absence of RANKL. Cell lysates were prepared, and detection of NFATc1 and PPARγ was determined by western blotting. Shown is a representative immunoblot from three experiments. Densitometric analyses of the level of RANKL (E) and PPARγ (F) were shown relative to the control (*n* = 3). *P <* *0.05 vs. control. ^#^ *P *<* *0.05 vs. RANKL. Rosi, rosiglitazone.

Previous reports demonstrated that PPARγ activation by pioglitazone or rosiglitazone inhibits RANKL‐induced osteoclastogenesis. As shown in Fig. 1A,B, our results also indicated that rosiglitazone treatment attenuated RANKL‐stimulated osteoclast differentiation in a dose‐dependent manner. Cytotoxicity of rosiglitazone was not observed on days 1 or 5 with the range of concentrations used (Fig. 1C). However, treatment with 5 μm of rosiglitazone reduced the level of RANKL‐induced NFATc1 abundance by about 40% without reducing PPARγ abundance (Fig. 1D–F). Rosiglitazone alone did not affect the level of either PPARγ or NFATc1.

### Effect of rosiglitazone on NFATc1 nuclear translocation

**Figure 2:**
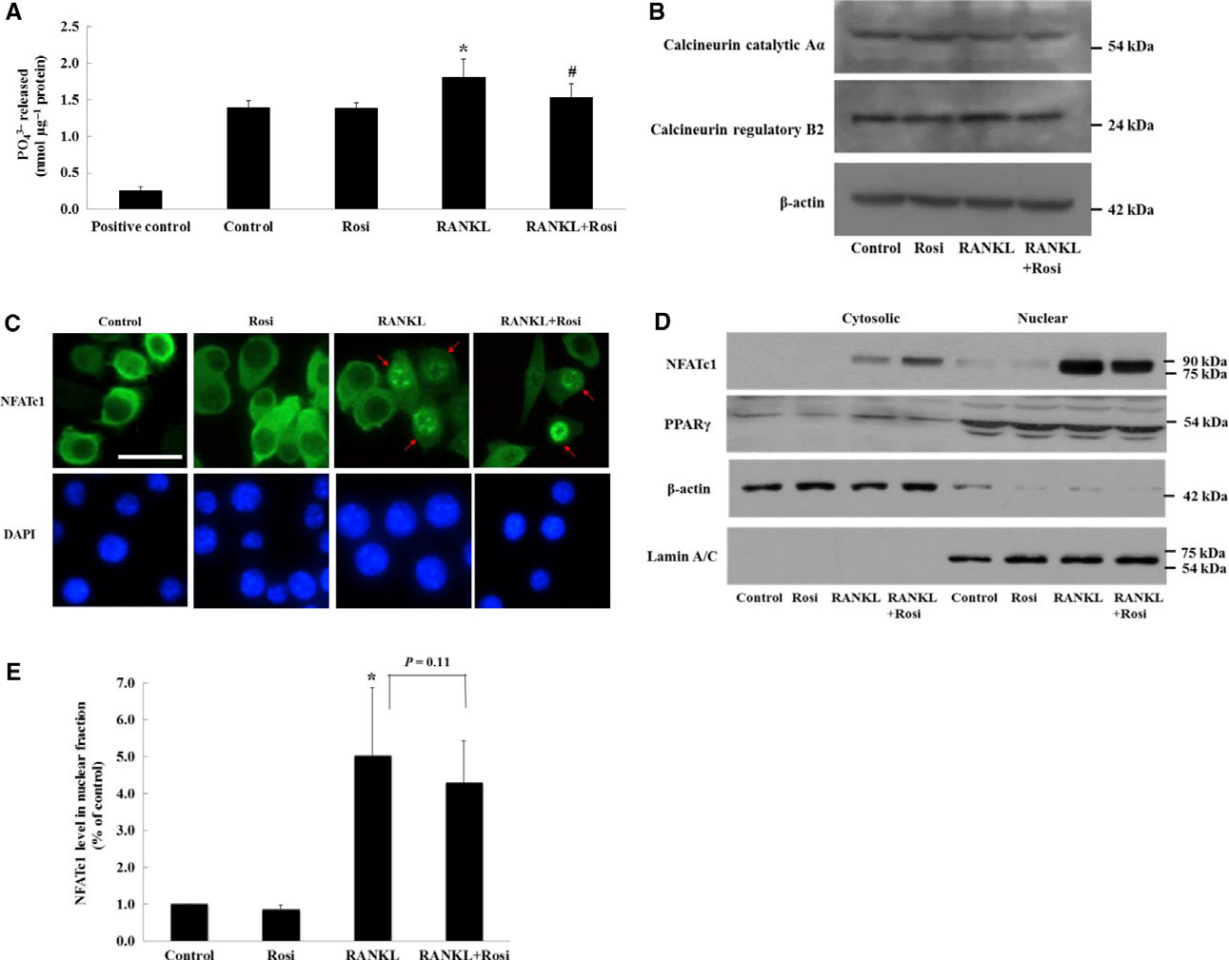
Rosiglitazone blocks NFATc1 nuclear import induced by RANKL. Cells were cultured for 24 h with the addition of 10 μm rosiglitazone in the presence of RANKL (50 μg·mL^−1^). (A) Ca^++^/calmodulin‐dependent calcineurin activity of cell lysates was measured in duplicate from three experiments. *P <* *0.05 vs. control. ^#^ *P *<* *0.05 vs. RANKL. (B) Protein levels of calcineurin isoforms in cell lysates were shown using anti‐calcineurin Aα (catalytic subunit A isoform α) and anti‐calcineurin B2 (regulatory subunit B type 2). (C) Immunofluorescence image for NFATc1 localization obtained using anti‐NFATc1 (green). Arrows indicate NFATc1 nuclear translocation. Scale bar, 20 μm. (D) Cytosolic and nuclear fractions were prepared for localization of NFATc1 and PPARγ. Equal amounts (10 μg) were loaded in each fraction. The image is representative of three experiments. (E) Based on (D), relative NFATc1 levels in the nuclear fraction were calculated through densitometric analyses (*n* = 3). *P <* *0.05 vs. control.

Nuclear factor of activated T cells, cytoplasmic 1 exhibits strong phosphorylation of serine residues in the resting state of cells. NFATc1 is initially dephosphorylated by the protein phosphatase calcineurin, which is a serine/threonine protein phosphatase that is composed of a catalytic A subunit and regulatory B subunit. Dephosphorylated NFATc1 is then translocated into the nucleus. Thus, we examined whether suppression of RANKL‐induced NFATc1 expression was due to blockage of NFATc1 nuclear translocation. As shown in Fig. 2A, RANKL stimulation moderately increased the activity of calcineurin compared with the control, and rosiglitazone reduced this increase. However, protein levels of calcineurin Aα and calcineurin B2 were not altered regardless of treatment with RANKL and/or rosiglitazone (Fig. 2B). Because changes in calcineurin activity can be accompanied by alterations in NFATc1 localization, both immunofluorescence microscopy and western blotting of the nuclear fraction were carried out. Immunofluorescence images revealed that rosiglitazone inhibited RANKL‐induced NFATc1 nuclear translocation (Fig. 2C). NFATc1 levels in the nuclear fraction showed similar alterations (Fig. 2D), although these findings were not statistically significant (Fig. 2E, *P* = 0.11). Meanwhile, most PPARγ remained in the nuclear fraction and its protein level was unchanged despite rosiglitazone treatment (Fig. 2D). Collectively, these findings imply that rosiglitazone inhibits NFATc1 nuclear translocation by suppressing the action of calcineurin activated by RANKL.

### Physical interaction between PPARγ and NFATc1

**Figure 3:**
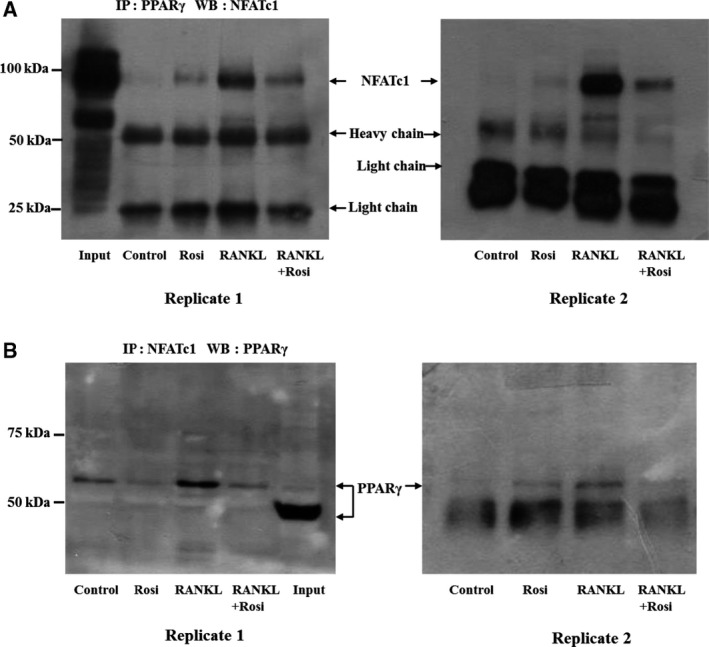
Rosiglitazone disrupts the physical interaction between PPARγ and NFATc1. After cells were cultured in the same fashion as described in Fig. 2, whole cell lysates were prepared and (A) immunoprecipitated with polyclonal anti‐PPARγ antibody and the NFATc1‐PPARγ complex was detected with monoclonal anti‐NFATc1 antibody or (B) immunoprecipitated with monoclonal anti‐NFATc1 antibody and the NFATc1‐PPARγ complex was detected with polyclonal anti‐PPARγ antibody. Two replicates are shown, but input control was not loaded in the second replicate of either (A) or (B).

Stimulation of steroid receptors by ligands disturbs NFAT binding to NFAT sites on DNA by complexing NFAT with steroid receptors 27. To test whether PPARγ activation has similar mechanisms, we analyzed the protein–protein interaction between PPARγ and NFATc1 *in situ*. As shown in Fig. 3, PPARγ‐NFATc1 binding was barely detectable under unstimulated conditions, and rosiglitazone alone did not affect this complex formation. However, RANKL strongly induced formation of the PPARγ‐NFATc1 complex, which was found in immunoprecipitation using either anti‐NFATc1 or anti‐PPARγ, and this induction was greatly attenuated by rosiglitazone. This indicates that ligand‐bound PPARγ disrupts the physical interaction between NFATc1 and PPARγ.

### Effects of PPARγ knockdown on NFATc1 mRNA expression and NFATc1 autoregulation

**Figure 4:**
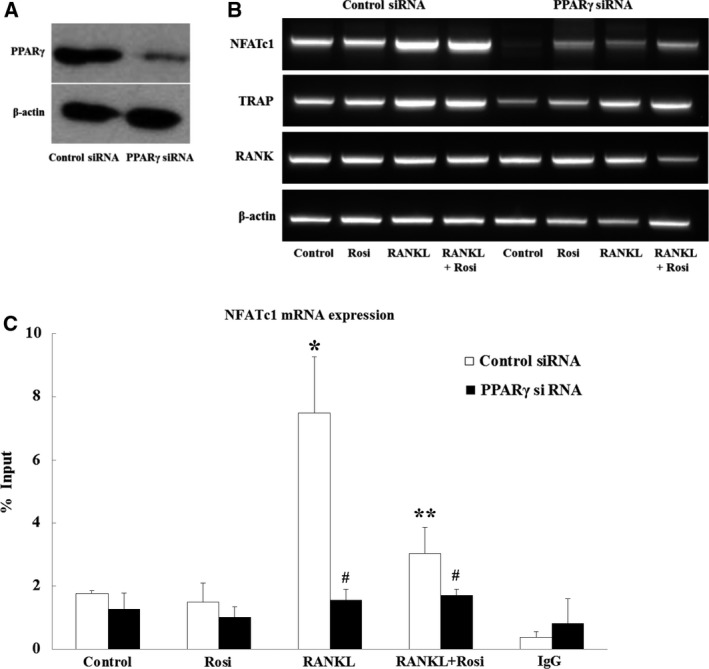
PPARγ knockdown inhibits mRNA expression of RANKL‐induced NFATc1 and inhibits RANKL‐induced NFATc1 binding to its own promoter. Cells were transfected with control siRNA or PPARγ siRNA, followed by incubation with rosiglitazone (10 μm) for 24 h in the presence or absence of RANKL. (A) PPARγ knockdown was confirmed by western blotting. (B) End‐point RT‐PCR was carried out under control siRNA and PPARγ siRNA to evaluate mRNA expression of the NFATc1‐dependent genes *NFATc1* and *TRAP* and the NFATc1‐independent genes *RANK* and *β‐actin*. The image is representative of two experiments. (C) After *in situ* ChIP assay, quantitative PCR was performed and levels of NFATc1 mRNA bound to the promoter were calculated. ChIP data are pooled from six measurements of two experiments. Statistical analysis was performed via two‐way ANOVA. *P <* *0.05 vs. control siRNA. **P <* *0.05 vs. RANKL. ^#^ *P *<* *0.05 vs. matched control siRNA.

To further explore the effects of PPARγ‐NFATc1 complex formation on positive autoregulatory NFATc1 expression, we performed a PPARγ knockdown experiment. When cells were transfected with PPARγ siRNA, PPARγ protein was barely expressed (Fig. 4A). As shown in Fig. 4B, knockdown of PPARγ significantly abolished mRNA expression of NFATc1 compared to levels seen with control siRNA, but knockdown barely affected the mRNA transcription of *RANK*, an NFATc1‐independent gene. It should be noted that the effect on PPARγ‐dependent expression of *TRAP* mRNA (TRAP, an NFATc1‐dependent osteoclast‐specific gene), although visible, was less profound than the effect on *NFATc1* mRNA expression. *TRAP* transcription can be also subject to regulation by RANKL‐mediated molecules, such as reactive oxygen species (ROS) and MITF transcription factor, which are physically unassociated with PPARγ 28, 29. These findings indicate that PPARγ‐NFATc1 complex formation is critical for autoregulatory transcription of the NFATc1 gene and expression of NFATc1‐dependent genes. To test whether transcriptional autoregulation of NFATc1 is followed by NFATc1 binding to its own promoter, we performed a ChIP assay. As depicted in Fig. 4C, quantitative PCR from the ChIP assay showed that rosiglitazone significantly blocked NFATc1 binding to its promoter despite RANKL stimulation, whereas in the absence of PPARγ, NFATc1 binding was completely prevented, regardless of RANKL stimulation.

## Discussion

Evidence indicates that PPARγ activation by natural ligands or synthetic drugs suppresses RANKL‐induced osteoclastogenesis by inhibiting NFATc1 expression 20, 21. We showed that rosiglitazone significantly attenuated osteoclast differentiation and the induction of NFATc1 expression by RANKL (Fig. 1). Because the NFATc1 transcription factor is a critical regulator in RANKL‐stimulated osteoclastogenesis, we further explored the molecular mechanism by which NFATc1 expression was inhibited by PPARγ activation. NFATc1 is autoamplified during the early phase of osteoclastogenesis 4, 11, 12. Prior to NFATc1 autoamplification, NFATc1 nuclear translocation is conventionally held to initially occur via calcineurin‐mediated dephosphorylation of NFAT. In our experiments, because protein levels of calcineurin subunits Aα and B2 were unchanged in treated groups compared with the control, RANKL‐induced increases in calcineurin activity are presumed to play certain roles in NFATc1 entry into the nucleus, which was to some degree inhibited by rosiglitazone. This was reflected by the reduced degree of NFATc1 nuclear translocation observed in immunofluorescence images and the lower NFATc1 protein level in the nuclear fraction after western blotting (Fig. 2C,D). Similar to our findings, Bao *et al*. found that rosiglitazone prevents endothelin‐1‐induced increases in both the activity and protein level of calcineurin in cardiomyocytes 30. Sander *et al*. also showed that rosiglitazone antagonized vascular endothelial growth factor (VEGF)‐mediated NFATc1 nuclear translocation in cardiac valve endothelial cells 31, but their study did not examine the action of calcineurin. On the other hand, Wagner *et al*. observed that PPARβ stimulation in neonatal cardiomyocytes directly induced calcineurin expression 32. It remains unclear whether PPARγ affects either the activity or protein level of calcineurin in a cell type‐specific or PPAR isoform‐specific manner.

Aside from the action of PPARγ activation on calcineurin, the reduction in RANKL‐induced NFATc1 expression by rosiglitazone could be influenced by changes in signaling pathways upstream of NFATc1 or modulatory actions of other transcriptional factors on NFATc1. Although a predicted PPAR response element (PPRE) is found in the NFATc1 promoter, direct transcriptional repression of PPARγ on NFATc1 expression is not possible because rosiglitazone alone did not affect NFATc1 abundance (Fig. 1). PPARγ negatively regulates gene expression through several transrepression mechanisms. One such mechanism is through the interaction of PPARγ with transcription factors such as NF‐κB, AP‐1, and NFAT 33, 34. In this regard, Yang *et al*. reported that the PPARγ agonist troglitazone increases formation of a complex between PPARγ and NFATc1 in PMA/ionomycin‐activated T cells 35. Similarly, Bao *et al*. demonstrated that rosiglitazone greatly enhances association of PPARγ with NFATc4 in endothelin‐1‐stimulated cardiomyocytes 30. We unexpectedly found that formation of an NFATc1‐PPARγ complex was strongly induced by RANKL stimulation and was reduced to basal levels by rosiglitazone treatment (Fig. 3). This implies that once bound or activated by ligands, PPARγ can be separated from the NFATc1‐PPARγ complex rather than remaining associated with NFATc1. Therefore, if PPARγ was absent or liganded within cells, NFATc1 would either remain alone or be associated with other transcriptional partners even upon RANKL stimulation. This hypothesis was partly confirmed by our PPARγ knockdown experiment. Knockdown of PPARγ resulted in suppression of mRNA expression of *NFATc1* and NFATc1‐dependent *TRAP*, an osteoclast‐specific gene, without affecting mRNA expression of NFATc1‐independent *RANK* (Fig. 4B). In addition, our ChIP experiment showed that PPARγ knockdown completely inhibited NFATc1 from binding to the NFAT site in its own promoter, regardless of RANKL stimulation (Fig. 4C). Taken together, when the NFATc1‐PPARγ complex is dissociated by PPARγ activation, NFATc1 seems to lose its binding affinity to NFAT sites, thereby lessening the transcriptional activity of NFATc1 that regulates its autoamplification and the mRNA expression of NFATc1‐dependent genes. Because both NFATc1 and NF‐kB share a core DNA binding motif (GGA), our findings are consistent with the molecular mechanical explanation suggested by Wen *et al*. 36. According to their hypothesis, PPARγ activation decreases the protein level of tumor necrosis factor‐α‐stimulated RANTES in kidney mesangial cells because liganded PPARγ dissociates from the NF‐κB‐PPARγ complex, inactivating the ability of NF‐κB to bind to the κB site in the RANTES promoter, thereby blocking RANTES expression. Therefore, we presume that dissociation rather than association of the NFATc1‐PPARγ complex plays an important role in suppression of NFATc1 expression.

In conclusion, PPARγ activation inhibits RANKL‐induced formation of an NFATc1‐PPARγ complex. Once dissociated from the complex, NFATc1 cannot bind to NFAT response elements in its own promoter, which suppresses the autoregulatory expression of NFATc1 during the early period of RANKL‐stimulated osteoclastogenesis.

## Author contributions

KLP, DGO, and DWA designed the study. DWA wrote the manuscript. KLP, DGO, YOK, and KSS performed the experiments. KLP, DGO, and DWA analyzed the data.
